# Anticancer natural products from the Middle East and North Africa: biodiversity, mechanisms, and translational challenges

**DOI:** 10.3389/fonc.2026.1846357

**Published:** 2026-07-06

**Authors:** Razane Hamiyeh, Zahraa Salhab, Hisham F. Bahmad, Antoine G. Abou Fayad, Wassim Abou-Kheir

**Affiliations:** 1 Department of Experimental Pathology, Immunology and Microbiology, Faculty of Medicine, American University of Beirut, Beirut, Lebanon; 2 Center for Drug Discovery, American University of Beirut, Beirut, Lebanon; 3 Department of Anatomy, Cell Biology and Physiological Sciences, Faculty of Medicine, American University of Beirut, Beirut, Lebanon; 4 Department of Pathology and Laboratory Medicine, University of Miami Miller School of Medicine, Miami, FL, United States

**Keywords:** anticancer mechanisms, cancer therapy, drug discovery, Middle East and North Africa (MENA), natural products

## Abstract

Cancer remains one of the leading causes of morbidity and mortality worldwide, imposing substantial clinical, societal, and economic burdens. Despite major advances in surgical oncology, systemic chemotherapy, radiation therapy, molecularly targeted therapeutics, and immune checkpoint inhibition, contemporary cancer treatment remains constrained by dose-limiting toxicities, intratumoral and intertumoral heterogeneity, and the inexorable emergence of multifaceted drug resistance mechanisms. These persistent therapeutic challenges have reinstated interest in natural products (NPs) as evolutionarily refined sources of anticancer agents characterized by structurally diverse molecular targets and pleiotropic mechanisms of action. Indeed, a substantial proportion of currently approved anticancer drugs are either directly derived from or structurally inspired by natural compounds. This comprehensive review examines the role of NPs as anticancer agents, with particular emphasis on bioactive compounds isolated from plants, fungi, marine organisms, and environmental bacteria indigenous to the Middle East and North Africa (MENA) region. We summarize exemplary MENA-derived NPs demonstrating cytotoxic, antiproliferative, pro-apoptotic, anti-angiogenic, anti-metastatic, and immunomodulatory activities across a wide range of preclinical cancer models. Mechanistically, these compounds converge on critical oncogenic signaling networks, including p53-caspase apoptotic cascades, NF-κB transcriptional inhibition, reactive oxygen species modulation, cell-cycle arrest, epigenetic reprogramming, and suppression of tumor invasion and chronic inflammation. In parallel, we highlight transformative technological innovations—including high-throughput phenotypic and biochemical screening platforms, metagenomics, genome mining algorithms, biosynthetic gene cluster activation, and synthetic biology approaches—that are fundamentally reshaping NP discovery and enabling access to previously cryptic or unculturable microbial biosynthetic pathways. These methodological advances, coupled with multi-omics integration, artificial intelligence-driven compound prediction, and heterologous expression systems, are accelerating the identification and characterization of structurally novel anticancer agents. Collectively, the evidence presented underscores the MENA region as a significantly underexplored yet exceptionally promising biodiverse reservoir of anticancer NPs with substantial therapeutic potential. Strategic harnessing of this biodiversity through interdisciplinary collaborative research, ethically governed bioprospecting frameworks, and translational development pipelines may yield structurally innovative, mechanistically distinct, and potentially safer therapeutic modalities to complement existing cancer treatments and address critical unmet clinical needs in precision oncology.

## 1 Introduction

The term “cancer” encompasses a heterogeneous spectrum of diseases unified by the uncontrolled proliferation of abnormal cells driven by the dysregulated acquisition of hallmark biological capabilities including sustained proliferative signaling, evasion of growth suppressors, and resistance to cell death (1, 2). Invasion of adjacent tissues and metastasis to distant sites represent advanced capabilities that may develop during tumor progression, but are not universally present at all stages or in all cancer types (3, 4). Characterized by dysregulated cell proliferation that evades endogenous homeostatic control mechanisms, cancer imposes the highest disease burden globally in terms of cause-specific disability-adjusted life years (DALYs), ranking second only to cardiovascular diseases among all causes of death (5, 6).

The global cancer burden is escalating rapidly, driven by demographic transitions including population shifts in the prevalence and distribution of modifiable risk factors associated with socioeconomic development and globalization (7). According to the most recent Global Burden of Disease Study 2023, there were 18.5 million new cancer cases and 10.4 million cancer deaths globally in 2023, contributing to 271 million DALYs (6). Between 1990 and 2023, new cancer cases increased by 105.1% and cancer deaths by 74.3%, although age-standardized mortality rates decreased by 23.9%, reflecting modest improvements in case-fatality rates (6). Projections indicate that by 2050, annual cancer incidence will reach 30.5 million cases and 18.6 million deaths, representing 60.7% and 74.5% increases from 2024 levels, respectively (6, 8). While cancer incidence rates are currently higher in high-income countries (HICs), the trajectory is markedly less favorable in low- and middle-income countries (LMICs). Between 1990 and 2023, age-standardized incidence rates decreased by 3% in HICs while increasing by 29% in lower-middle-income and 24% in low-income countries; age-standardized mortality rates declined by 27% in HICs but increased by 17% and 14% in lower-middle-income and low-income countries, respectively (6). This divergence reflects structural disparities specific to LMICs, including a higher proportion of infection-attributable cancers, notably cervical, hepatic, and gastric cancers linked to HPV, HBV, and *H. pylori* (9), alongside elevated mortality-to-incidence ratios driven by late-stage presentation, limited population-based cancer registry infrastructure, and restricted access to radiotherapy and systemic therapies (10, 11). In addition, forecasted mortality increases of 90.6% in LMICs compared to 42.8% in high-income countries reflect profound disparities in healthcare infrastructure, diagnostic capacity, and therapeutic access, underscoring the urgency of addressing these region-specific vulnerabilities (12, 13).

The economic burden not only includes direct medical costs but also extends to indirect costs from lost income due to long-term disability and caregiving responsibilities, which severely impact household finances and national economies (14). In the United States alone, annual cancer-attributable healthcare expenditures were estimated at $208.9 billion in 2020, with costs projected to escalate sharply due to the proliferation of expensive molecularly targeted therapies and expanding cancer survivor populations. Productivity losses from cancer-related mortality contribute an additional $30 billion annually. In LMICs, the economic burden extends beyond direct medical costs to encompass indirect costs from prolonged disability, lost income, and caregiving responsibilities, profoundly impacting household finances and national economic productivity (15).

**Figure 1:**
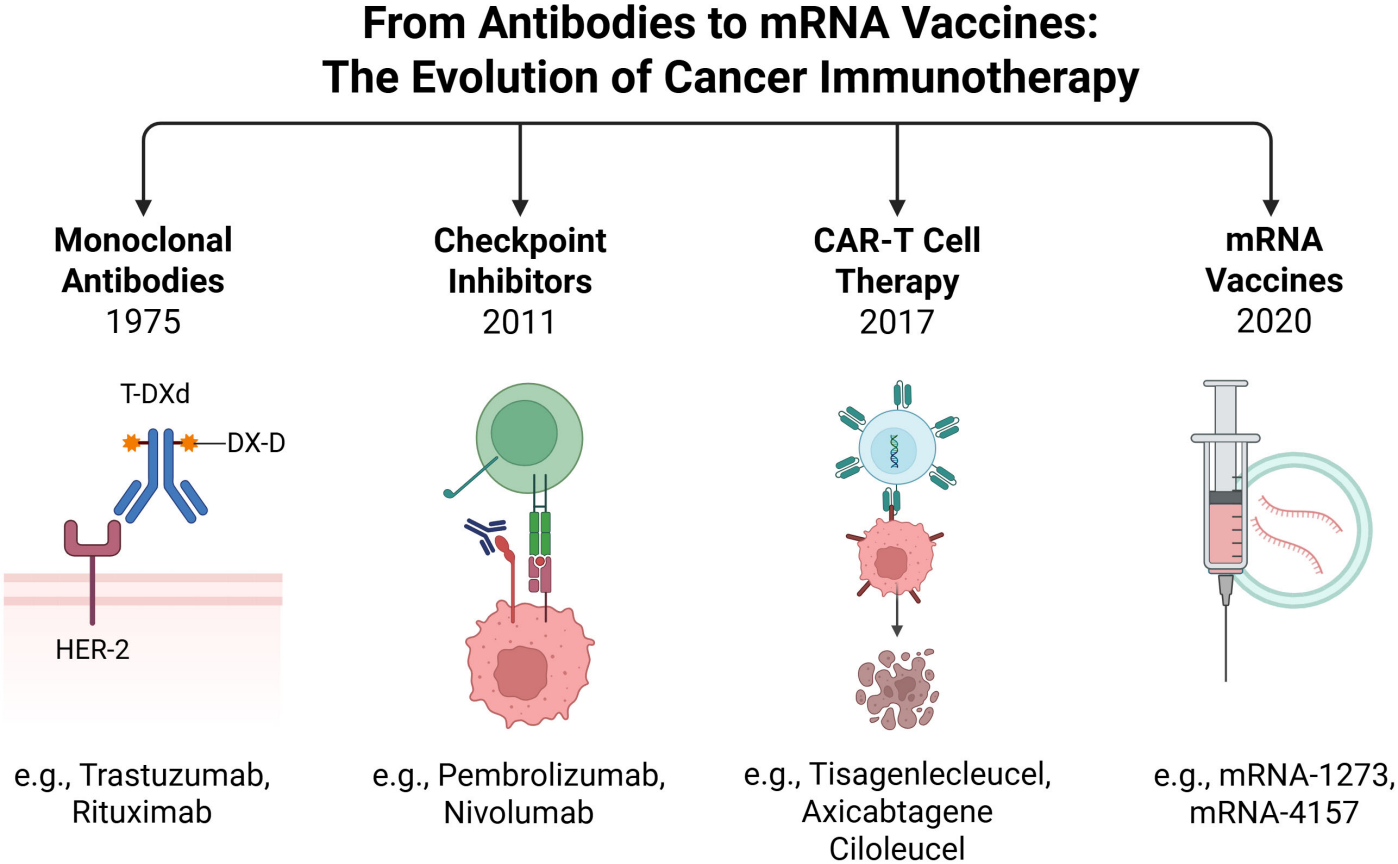
Chronological evolution of major cancer treatment modalities from surgical resection to modern targeted and immunotherapeutic approaches. Created in BioRender. Bahmad, H (2026). https://BioRender.com/137o111.

**Figure 2:**
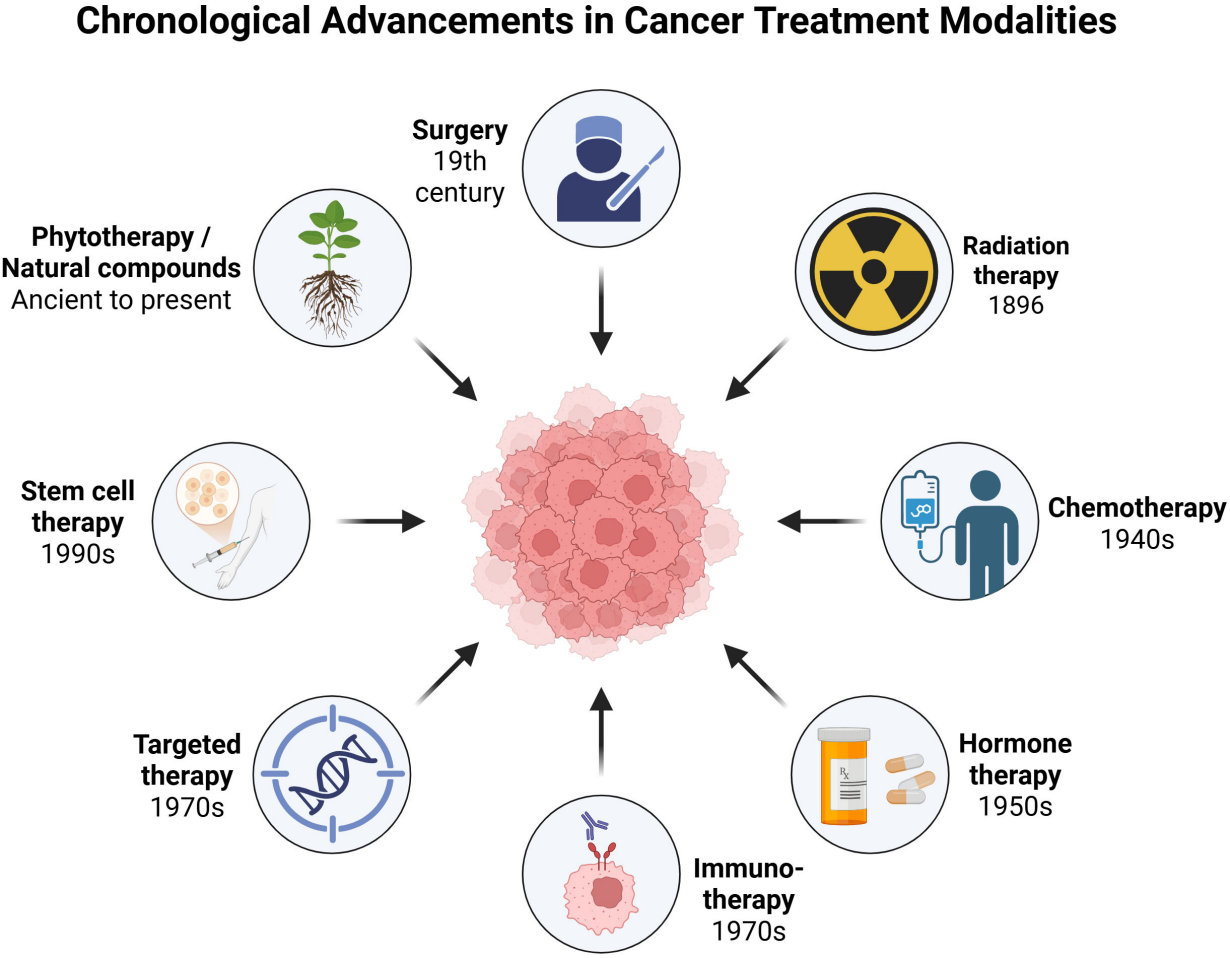
Evolution of cancer immunotherapy highlighting key milestones from monoclonal antibodies to checkpoint inhibitors, CAR-T cell therapy, and mRNA-based vaccines. Created in BioRender. Bahmad, H (2026). https://BioRender.com/yfhyqge.

Given the complex and rapidly evolving nature of cancer treatment, multimodal therapeutic strategies have been developed including surgical resection, radiotherapy, systemic chemotherapy, molecularly targeted therapy, and immunotherapy (16) (**Figures 1**, **2**). The selection of therapeutic modality depends on tumor type, stage, molecular characteristics, and patient-specific factors. Surgery remains the primary curative approach for localized, non-disseminated primary tumors (17). Radiotherapy is predominantly utilized in localized settings, often in conjunction with surgical intervention, and plays a critical role in palliative care (18). Yet, systemic chemotherapy remains the cornerstone treatment for both solid and hematologic malignancies, employing cytotoxic agents to inhibit cancer cell proliferation (19). However, conventional chemotherapy is constrained by significant dose-limiting toxicities attributable to non-selective cytotoxic effects on both malignant and rapidly dividing normal cells, profoundly compromising quality of life (20–23). Beyond toxicity, treatment resistance represents an equally formidable obstacle. Resistance is mechanistically multifactorial, driven not only by intratumoral heterogeneity but also by cellular plasticity — encompassing epithelial-to-mesenchymal transition, cancer stem cell acquisition, and transcriptional reprogramming under therapeutic pressure — as well as clonal evolution, whereby treatment itself acts as a Darwinian selective force favoring the expansion of pre-existing resistant subclones [A, B]. These overlapping mechanisms collectively limit the durability of response across treatment modalities and highlight the need for agents capable of engaging multiple oncogenic pathways simultaneously (24).

A paramount challenge limiting therapeutic efficacy is the emergence of chemoresistance, a multifaceted phenomenon wherein cancer cells evolve mechanisms to evade cytotoxic effects of drugs, culminating in treatment failure and disease relapse (22, 25, 26). Chemoresistance mechanisms are multifactorial and include intratumoral heterogeneity, wherein specific subpopulations within a tumor, especially cancer stem cells (CSCs), acquire intrinsic resistance to chemotherapeutic agents (27, 28). CSCs are characterized by self-renewal capacity, resistance to apoptosis, enhanced DNA repair machinery, and overexpression of ATP-binding cassette (ABC) transporters (particularly ABCB1, ABCG2, and ABCC1), which actively efflux chemotherapeutic agents from cells, reducing intracellular drug concentrations and therapeutic efficacy (29–32). This multidrug resistance (MDR) phenotype is particularly prevalent in leukemia, breast, ovarian, and colorectal cancers (30, 32, 33). Additional resistance mechanisms include activation of prosurvival signaling pathways (PI3K/Akt, Wnt/β-catenin, Notch, Hedgehog), epithelial-mesenchymal transition (EMT), enhanced DNA damage response, apoptosis evasion, and metabolic reprogramming (22, 34). Therefore, drug resistance occurrence in different tumors is jeopardizing their efficacy, and any improvement in the outcomes has to involve new approaches that can circumvent these mechanisms and enhance patient resilience against relapse while maintaining enhanced efficacy, reduced toxicity, and economic accessibility (35). A comparative overview of the relative advantages and limitations of natural products versus synthetic agents in cancer therapy is presented in **Table 1**.

**Table 1:** Comparative advantages of NPs versus synthetic drugs in cancer therapy.

Feature	NPs	Synthetic drugs	References
Complexity of Structure	High molecular complexity, often with unique scaffolds and diverse functional groups that enhance bioactivity	Generally simpler structures, which may limit interaction with complex cancer targets but allows easier synthesis	(2, 36)
Selectivity and Targeting	Often exhibit high selectivity for cancer cells due to interaction with multiple molecular targets	Typically target single pathways, which can lead to drug resistance and lower selectivity for cancer cells	(37, 38)
Side Effects	Generally lower side effects as they tend to be more selective, impacting fewer normal cells	Higher incidence of side effects due to broader impact on normal cells and non-specific targeting	(23, 39)
Resistance Development	Lower rates of resistance development, attributed to complex multi-target interactions	High potential for resistance development, especially in drugs targeting single pathways or mechanisms	(29, 33)
Toxicity	Typically, lower toxicity levels, particularly in compounds derived from dietary or traditional medicine sources	Potentially high toxicity, often necessitating dose adjustments and combined use with other drugs	(40, 41)
Source Availability	Derived from natural sources like plants, fungi, and marine organisms, which can be renewable and sustainable	Manufactured synthetically, enabling mass production but often resource-intensive and costly to develop	(42, 43)
Development Challenges	Complex extraction and purification processes; can be difficult to isolate in sufficient quantities	Easier to develop and modify in labs, but may lack the structural diversity of NPs	(44, 45)
Discovery Potential	High potential for discovering novel mechanisms and pathways through diverse bioactivity	Limited diversity and often focused on known chemical libraries, reducing novel discovery potential	(36, 46)
Regulatory Approval	Often requires additional safety testing due to complex mixtures and new sources	Generally streamlined regulatory approval with standardized synthetic processes and known safety profiles	(47, 48)

Thus, cancer control has become a global health priority calling for innovative approaches to prevention, early detection, and treatment, including exploring natural products (NPs) as evolutionarily refined sources of anticancer agents characterized by structurally diverse molecular scaffolds and pleiotropic mechanisms of action (14, 36, 42). Natural products have been integral to traditional medicine systems for years and continue to play a crucial role in modern drug discovery, particularly in cancer therapy. Remarkably, many anticancer drugs currently in clinical use, such as paclitaxel, vincristine, etoposide, and doxorubicin are derived from natural sources (*Taxus brevifolia*, *Catharanthus roseus*, *podophyllotoxin*, and *Streptomyces* species, respectively) (2, 37, 49–52). In fact, of the 185 small-molecule anticancer agents approved between January 1981 and September 2019, only 15.7% were purely synthetic compounds, while the remaining ~84% were classified as natural products, natural product derivatives, or synthetics structurally inspired by natural product scaffolds, spanning categories N (unmodified natural products), NB (natural product botanicals), ND (natural product derivatives), S* (synthetics with a natural product pharmacophore), S*/NM, and S/NM (natural product mimics) (2). Natural compounds are often structurally diverse and possess unique bioactive properties that allow them to target multiple molecular pathways implicated in cancer progression, including dysregulated cell proliferation, apoptosis evasion, metastasis, and immune evasion (36, 50, 52–54). By exploring the vast chemical diversity of NPs, researchers can identify structurally novel lead compounds with potential to offer mechanistically distinct and potentially safer therapeutic options for cancer patients.

Given the pressing need for innovative cancer treatments that address both efficacy and safety limitations of conventional therapeutics, this review aims to evaluate the potential of NPs, particularly those derived from plants, fungi, marine organisms, and environmental bacteria indigenous to the Middle East and North Africa (MENA) region, as sources of novel anticancer agents. Although NPs have been central to cancer drug discovery worldwide, the MENA region remains relatively underexplored, with limited systematic research focused on uncovering bioactive compounds from its unique biodiversity. Critical questions remain: How do these MENA-derived compounds interact with molecular pathways in cancer cells to inhibit growth, induce apoptosis, or prevent metastasis? Can they offer structurally innovative therapeutic options that circumvent chemoresistance mechanisms and reduce systemic toxicity? By advancing NP discovery for anticancer activity in this largely untapped region, this review aims to identify promising compounds, elucidate their mechanisms of action, and assess their translational potential in addressing unmet clinical needs. Through this focus, we underscore the strategic importance of the MENA region as an exceptionally promising reservoir of anticancer NPs with substantial potential to provide safer, more effective therapeutic alternatives for cancer patients globally.

## 2 Literature search strategy

A narrative literature review was conducted using three electronic databases: PubMed, Scopus, and Google Scholar. No date restrictions were applied. The following search terms were used, alone and in combination: “natural products, “ “secondary metabolites, “ “drug discovery, “ “anticancer, “ “antineoplastic, “ “plant-derived compounds, “ “marine natural products, “ “fungal metabolites, “ “bacterial natural products, “ and “Middle East and North Africa.” Only peer-reviewed articles published in English were included. For the general natural product sections, inclusion was restricted to compounds that have entered clinical trials or are currently approved for clinical use, ensuring translational relevance. For the MENA-specific sections, this criterion was broadened to include preclinical studies reporting cytotoxic or antineoplastic activity of compounds isolated from plants, marine organisms, fungi, or bacteria endemic to or collected from MENA countries, reflecting the comparatively limited clinical development of compounds from this region. Review articles, original research articles, and clinical trial reports were all considered eligible for inclusion. No formal meta-analysis or quantitative synthesis was performed, consistent with the narrative review methodology.

## 3 Overview of NPs

### 3.1 Definition and classification of NPs

Nature has served as a prolific source of therapeutic compounds for millennia and continues to provide structurally diverse chemotypes and pharmacophores with exceptional medicinal potential (55). As researchers seek innovative solutions to overcome chemoresistance and enhance therapeutic outcomes, NPs have emerged as a strategically important resource, offering bioactive compounds with unique mechanisms capable of counteracting cancer’s multifaceted defense strategies.

Natural products are bioactive compounds biosynthesized by living organisms, typically as components of their defense mechanisms or competitive survival strategies (56, 57). Compounds essential for an organism metabolism are termed metabolites, representing terminal products of metabolic pathways and typically comprising low-molecular-weight molecules present in numerous marine species, microorganisms, fungi, plants, and animals. Bioactive metabolites are categorized into primary and secondary metabolites based on their biosynthetic origin and functional roles (58–60).

Primary metabolites are microbial and plant products used for building cellular constituents and represent integral components of normal growth processes. They include intermediates and end‐products of anabolic metabolism or result from catabolic metabolism, directly participating in essential cellular functions such as respiration, growth, and development. In contrast, secondary metabolites (also termed specialized metabolites) are biosynthesized *de novo* from simple precursors of primary metabolism through elaborate enzymatic reaction sequences catalyzed by specific biosynthetic machinery (56, 57, 60, 61). Functionally distinct from primary metabolites, secondary metabolites comprise a diverse group of specialized small molecules that mediate defense responses, stress adaptation, and ecological communication (57, 59). These compounds possess remarkable structural and biological diversity and have proven to be potent sources of medicinal agents, particularly in oncology (56, 57, 62). The structural and pharmacokinetic properties relevant to the therapeutic application of NPs in cancer treatment are outlined in **Table 2**, and the major classes of NPs, together with their sources, principal mechanisms of action, and corresponding target cancers, are summarized in **Table 3**.

**Table 2:** Structural and pharmacokinetic properties of NPs in cancer treatment.

Feature	NPs	References
Complex Structural Diversity	NPs often possess complex structural scaffolds, which enhance their ability to interact with diverse biological targets. This structural diversity allows them to engage in multiple interactions within cancer pathways, often making them more effective than simpler synthetic compounds.	(2, 36)
Unique Mechanisms of Action	Many NPs target multiple pathways in cancer cells, offering mechanisms that are often novel and more intricate than those of synthetic drugs. For instance, compounds like taxanes and vinca alkaloids work by targeting microtubules, disrupting cell division in cancer cells.	(39, 63, 64)
Improved Selectivity	NPs frequently exhibit high selectivity for cancer cells, reducing damage to normal cells. This selectivity is partly due to their complex structures that interact specifically with cancer cell biomarkers.	(37, 38, 65)
Fewer Side Effects	Due to their selectivity, NPs like curcumin and ingenol mebutate often have fewer side effects compared to conventional chemotherapy agents, which tend to be non-selective and impact normal cells.	(66, 67)
Lower Resistance Development	NPs can engage multiple cellular pathways, which makes it more difficult for cancer cells to develop resistance compared to single-target synthetic drugs. Compounds like salinomycin target both cancer cells and cancer stem cells, reducing the likelihood of resistance.	(29, 68)
Enhanced Bioavailability	Many natural compounds have high bioavailability due to their lipophilic properties, facilitating better absorption and cellular uptake. Some, like marine-derived compounds, are known for their ability to penetrate biological membranes effectively.	(69–71)
Source Sustainability	NPs can often be sustainably sourced or derived from renewable natural resources, such as plants, marine organisms, or microbial sources. Techniques like metagenomics are being used to access these resources without harming the original sources.	(42, 44)

**Table 3:** Classes of NPs with sources, mechanisms of action, and target cancers.

Class of NP	Examples of NPs	Source	Mechanism of action	Target cancers	References
Plant-Derived Compounds	Paclitaxel, Vincristine, Camptothecin, Curcumin	Taxus brevifolia, Catharanthus roseus, Curcuma longa	Paclitaxel and vincristine disrupt microtubules; camptothecin inhibits topoisomerase I; curcumin inhibits NF-κB and VEGF	Breast, Ovarian, Lung, Colorectal	(2, 39, 64, 67, 72)
Fungal Compounds	Griseofulvin, Terrein, Brefeldin A, Gliotoxin	Penicillium notatum, Aspergillus terreus, Gliocladium spp.	Griseofulvin inhibits centrosomal clustering; terrein inhibits RhoB signaling; brefeldin A blocks Golgi apparatus partitioning	Breast, Prostate, Colorectal	(41, 73–75)
Marine-Derived Compounds	Halichondrin B, Didemnin B, Trabectedin, Lamellarin D	Marine sponges (e.g., Halichondria okadai), Caribbean tunicate, Lamellaria sp.	Halichondrin B disrupts microtubules; didemnin B inhibits protein synthesis; trabectedin binds DNA; lamellarin D inhibits topoisomerase I	Breast, Ovarian, Lung, Soft Tissue	(69, 76–79)
Microbial-Derived Compounds	Doxorubicin, Bleomycin, Mitomycin C, Salinomycin	Streptomyces peucetius, Streptomyces verticillus, Streptomyces caespitosus	Doxorubicin intercalates DNA; bleomycin induces DNA strand breaks; mitomycin C cross-links DNA; salinomycin targets cancer stem cells	Leukemia, Bladder, Lung, Breast	(2, 68, 80, 81)
Bacterial Symbiotic Compounds	Dolastatin, Psammaplin, Ecteinascidin	Marine cyanobacteria (e.g., Symploca sp.), Caribbean tunicate, Pseudomonas fluorescens	Dolastatin disrupts microtubules; psammaplin inhibits HDACs; ecteinascidin binds minor DNA groove	Leukemia, Lymphoma, Breast	(82–85)

### 3.2 Sources of NPs

The following sections examine the anticancer potential of NPs according to their biological source, highlighting representative compounds, their mechanisms of action, and their translational relevance in oncology.

#### 3.2.1 Non-bacterial derived NPs with antineoplastic activities

##### 3.2.1.1 Plants

Among diverse sources of NPs, plants have historically served as essential therapeutic resources and have been integral components of traditional medicine globally (86). Currently, in many developing countries, more than 70% of the population relies on medicinal plants and herbal remedies as primary healthcare interventions (40). With a well-documented repertoire of compounds demonstrating potent anticancer activity, plant extracts possess immense biological activities against a wide range of molecular targets implicated in cancer progression (37, 86).

This remarkable capacity of plants to eliminate malignant cells represents the culmination of extensive evolutionary processes. As plants evolved mechanisms to eliminate rapidly dividing infectious pathogens, they developed the capacity to biosynthesize secondary metabolites with cytotoxic properties against heavily proliferating organisms (38). These same NPs have proven therapeutically beneficial as antineoplastic agents. An extensive array of plant-derived NPs with anti-cancer activities has been discovered over recent decades.

Microtubule-targeting agents represent a major class that inhibits proliferation by disrupting mitotic spindle function (63). These molecules exert anticancer effects through diverse mechanisms: disrupting microtubule dynamics (podophyllotoxin and combretastatin A-4) (37, 87), stabilizing microtubules (taxanes including paclitaxel and docetaxel) (88), or exhibiting dose-dependent dual actions (*vinca* alkaloids, including vincristine and vinblastine, and colchicine) (37, 89). Mechanistically, these agents bind to distinct sites on tubulin heterodimers, such as the taxane-binding site, vinca-binding site, and colchicine-binding site, resulting in mitotic arrest, aberrant spindle formation, and subsequent apoptosis (90–93).

DNA topoisomerase inhibitors represent another critical class that induces cell death by restricting DNA repair machinery. These include camptothecin and its derivatives (topotecan, irinotecan), which form stabilized complexes with DNA and topoisomerase I (94), preventing DNA religation and inducing double-strand breaks (37, 49). Additionally, salvicine interacts with topoisomerase II, similarly disrupting DNA topology and triggering apoptotic cascades (95).

Apoptosis-targeting agents represent a diverse class of plant-derived compounds that promote programmed cell death through multiple pathways. These extracts enhance cell death by activating the extrinsic (death receptor) pathway (*Cudrania tricuspidata* stem extract) (65), the intrinsic (mitochondrial) pathway (geniposide) (96), or both pathways simultaneously (glycyrrhetinic acid) (97). These compounds modulate Bcl-2 family proteins, activate caspase cascades, induce cytochrome c release, and regulate death receptor signaling (53, 98, 99).

Protein synthesis inhibitors block ribosomal peptidyl transferase activity, exemplified by homoharringtonine (omacetaxine mepesuccinate, approved for chronic myeloid leukemia) and bruceantin (100, 101).

Cell cycle disruptors encompass a diverse class of plant-derived compounds that interfere with cell cycle progression through multiple mechanisms. Ellipticine, a pyrido[4, 3-b]carbazole alkaloid isolated from *Ochrosia* species, exhibits different modes of action encompassing DNA intercalation, topoisomerase II poisoning, covalent DNA adduct formation via cytochrome P450-mediated oxidation, and cell cycle arrest, all of which ultimately lead to apoptotic cell death (102, 103). Roscovitine, a purine analog, acts primarily as CDK inhibitor by competing with ATP at the conserved catalytic binding site, therefore blocking cell cycle progression at multiple checkpoints (102, 104). Its anticancer activity also extends to transcriptional suppression via RNA polymerase II inhibition and MYC downregulation, reflecting a broader polypharmacological profile (105).

Although these NPs and their semi-synthetic derivatives are extensively utilized in chemotherapeutic regimens nowadays, continuous discovery of novel and potent treatments remains imperative. Recent plant-derived compounds under investigation include ingenol mebutate (ingenol-3-angelate, PEP005) secreted by *Euphorbia* species (106), which exerts antitumor effects through multiple pathways including protein kinase C (PKC) modulation, primary necrosis induction, and immune system activation to eliminate residual malignant cells (66, 107). Moreover, curcumin, a polyphenol compound from *Curcuma longa* (67), has been extensively investigated for its potent anticancer actions mediated through targeting multiple processes including autophagic cell death promotion, NF-κB (Nuclear Factor kappa B) nuclear translocation inhibition, and VEGF (Vascular Endothelial Growth Factor) expression downregulation (72, 108).

##### 3.2.1.2 Fungi

Fungi, particularly filamentous species, offer another rich source of bioactive compounds with potent antineoplastic properties, often initially characterized for antimicrobial actions and subsequently repurposed for oncological applications (109). Following the discovery of penicillin from the filamentous fungus *Penicillium notatum* (41), extensive research has expanded to explore fungi as prolific sources of structurally diverse NPs with broad biological activity.

Representative fungal metabolites demonstrate remarkable mechanistic diversity. The polyketide terrein inhibits RhoB and Rac1 signaling, suppressing metastatic processes including cell adhesion, migration, and invasion, while simultaneously attenuating angiogenesis through VEGF/VEGFR2 pathway disruption and downstream PI3K/AKT/mTORC1 signaling inhibition (73).

Brefeldin A disrupts mitotic progression by inhibiting the fission machinery essential for Golgi apparatus partitioning during cell division (74).

Griseofulvin, beyond its established antifungal properties, demonstrates anticancer efficacy through suppression of microtubule dynamic instability and inhibition of centrosomal clustering, inducing G2/M cell cycle arrest at concentrations that kinetically suppress spindle microtubule dynamics without requiring complete depolymerization (110–113). Nano-engineered formulations of griseofulvin have demonstrated enhanced cellular uptake and activation of multiple apoptotic pathways including p53, Bax/Bcl-2, and caspase-3, while modulating VEGFR2 and pAKT signaling (112).

Beyond the mechanistically characterized compounds above, fungal-derived natural products of structurally heterogeneous classes, including cytochalasins, phomazines, diketopiperazines, and non-ribosomal peptides, further illustrate the chemical breadth and anticancer potential of fungal secondary metabolites. Penochalasins I and J isolated from *Penicillium chrysogenum* exhibited cytotoxic activity against MDA-MB-435 and SGC-7901 gastric cancer cell lines (114).

Phomazines A-C derived from *Phoma* sp. OUCMDZ-1847 demonstrated cytotoxicity across multiple hematologic and solid tumor cell lines including HL-60 leukemia and HCT-116 colorectal cancer cells (115).

TMC-264 from *Rhizopycnis vagum* showed potent antineoplastic activity against five human cancer cell lines (116).

Non-ribosomal peptides (NRPs) also contribute significantly to the fungal anticancer armamentarium: gliotoxin induces colorectal cancer cell death by downregulating Wnt signaling and β-catenin levels (75), while Leucinostatin A inhibits prostate cancer cell growth by reducing the expression of insulin-like growth factor-I (117).

##### 3.2.1.3 Marines

The marine environment’s unique ecosystems, including intense spatial competition at reef fringes and deep-sea vents (69), drive organisms to biosynthesize potent secondary metabolites as chemical weapons for territorial defense and predation, many of which demonstrate remarkable antineoplastic properties (70, 118, 119). This biodiversity has yielded several clinically approved anticancer agents and numerous candidates in advanced development.

Didemnin B, a cyclic depsipeptide originally isolated from the Caribbean tunicate *Trididemnum solidum*, represents one of the first marine NPs to advance to phase II clinical trials. This compound possesses broad-spectrum antitumor activity through multiple mechanisms: inhibition of DNA, RNA, and protein synthesis; non-competitive binding to palmitoyl protein thioesterase; and induction of apoptosis mediated by FK506-binding protein 25 (FKBP25) (76, 120–123).

Eribulin mesylate, a synthetic analogue of halichondrin B isolated from the marine sponge *Halichondria okadai*, exemplifies successful clinical translation of marine NPs. Approved for metastatic breast cancer and liposarcoma, eribulin functions as a non-taxane microtubule dynamics inhibitor that binds specifically to microtubule plus ends, suppressing polymerization without affecting depolymerization and inducing characteristic G2/M cell cycle arrest (69, 77, 124). Beyond its antimitotic effects, eribulin demonstrates unique non-mitotic mechanisms including tumor vasculature remodeling, increased vascular perfusion, reduced hypoxia, and reversal of epithelial-to-mesenchymal transition (EMT), collectively contributing to improved overall survival in clinical trials (125, 126).

Trabectedin (ecteinascidin-743, ET-743), originally isolated from the sea squirt *Ecteinascidia turbinata* but subsequently identified as a product of symbiotic *Pseudomonas fluorescens*, represents a paradigm of marine-microbial natural product discovery. This tetrahydroisoquinoline alkaloid binds the DNA minor groove, inducing unprecedented bending toward the major groove and disrupting transcription-coupled nucleotide excision repair and homologous recombination pathways (127–130). Trabectedin is approved for advanced soft tissue sarcoma and relapsed platinum-sensitive ovarian cancer in combination with pegylated liposomal doxorubicin (131). Its mechanism extends beyond direct DNA damage to include inhibition of activated transcription through RNA polymerase II interaction and degradation, modulation of tumor microenvironment through effects on tumor-associated macrophages, and displacement of oncogenic transcription factors from target promoters (127).

Lurbinectedin, a synthetic trabectedin analogue featuring a tetrahydro-β-carboline moiety replacing the tetrahydroisoquinoline C subunit, was developed to enhance activity and reduce toxicity. Approved by the FDA in 2020 for metastatic small cell lung cancer (132), lurbinectedin operates through similar DNA-binding mechanisms while demonstrating particular efficacy in BRCA-mutated and homologous recombination-deficient tumors, potentially mediated by immune-modulating mechanisms (129, 130, 133).

Lamellarin D, isolated from marine mollusk of the *Lamellaria* genus, functions as highly cytotoxic topoisomerase I inhibitor (78, 134, 135). Additional marine-derived compounds in clinical development include antibody-drug conjugates utilizing auristatin payloads (ladiratuzumab vedotin, glembatumumab vedotin for triple-negative breast cancer; disitamab vedotin for HER2-positive disease) and SGN-CD228A, currently in phase I trials for various solid tumors.

#### 3.2.2 Bacterial-derived NPs with antineoplastic activities

Certain bacteria, particularly those from marine and soil environments, biosynthesize complex molecules to survive harsh conditions, many of which exhibit promising anticancer properties and provide a new avenue for cancer therapy development.

##### 3.2.2.1 Marine bacteria

Accumulating evidence demonstrates that many compounds initially attributed to marine invertebrates originate from symbiotic or commensal bacteria, which synthesize these molecules to provide protective or predatory advantages to their hosts (37, 69, 136). This shift has profound implications for sustainable production and mechanistic understanding.

The dolastatin family exemplifies this phenomenon. Dolastatin 10 and 15, originally extracted from sea hares (*Aplysia* species), were subsequently traced to cyanobacterial symbionts through genomic analysis. These peptides disrupt mitotic cell division at nanomolar concentrations across multiple solid tumor types (82, 137–141). Psammaplins, including Psammaplin A and its dimer bisaprin, were initially isolated from verongid sponges (*Psammaplysilla* sp.) but later confirmed to originate from associated cyanobacteria and heterotrophic bacteria. These agents demonstrate cytotoxicity against human endometrial Ishikawa cancer cells through dual inhibition of histone deacetylases (HDACs) and DNA methyltransferases, targeting epigenetic regulatory mechanisms (83, 142).

Ecteinascidin (trabectedin), discussed above, represents another misattributed marine molecule, with biosynthetic gene clusters identified in *Pseudomonas fluorescens* rather than the tunicate *Ecteinascidia turbinata*. Several dolastatin-derived antibody-drug conjugates have advanced to late-stage clinical development, including SGN-CD228A (phase I for solid tumors), brentuximab vedotin with disitamab for non-small cell lung cancer (phase II), and mafodotin with depatuxizumab for glioblastoma (phase III) (84, 85, 143–149).

##### 3.2.2.2 Soil-dwelling bacteria

Soil-dwelling bacteria, particularly *Streptomyces* species, have yielded the majority of clinically utilized bacterial anticancer agents (150–152). Doxorubicin (DOXO), an anthracycline antibiotic produced through genetic modification of *Streptomyces peucetius*, exemplifies this class. DOXO exerts antitumor effects through multiple mechanisms including DNA intercalation, topoisomerase II inhibition, free radical generation, and direct membrane effects. Despite its broad-spectrum activity and favorable therapeutic index, significant cardiotoxicity necessitates ongoing formulation optimization. Non-pegylated liposomal formulations (Nudoxa^®^) have advanced to phase III clinical trials, demonstrating improved targeted delivery and reduced systemic toxicity (80, 153).

Bleomycin, derived from *Streptomyces verticillus*, represents a unique glycopeptide antibiotic requiring Fe(II) for activity. The Fe(II)-bleomycin complex binds guanosine-cytosine-rich DNA regions and generates highly reactive free radicals that produce DNA single-strand breaks at 3’-4’ deoxyribose bonds, yielding free base propenals and inducing G2-phase-specific cytotoxicity (154–158). Bleomycin demonstrates particular efficacy against Hodgkin lymphoma, testicular cancer, and head and neck malignancies, though therapeutic application is limited by dose-dependent pulmonary fibrosis. Next-generation sequencing analysis of over 200 million double-strand breaks revealed expanded sequence specificity at 5’-RTGTAY motifs in cellular DNA versus 5’-TGTAT in purified DNA, suggesting microenvironmental influences on drug-DNA interactions (156).

Mitomycin C, derived from *Streptomyces caespitosus*, functions as a bioreductive alkylating agent requiring enzymatic or chemical reduction for activation. Upon reduction, mitomycin C undergoes nucleophilic attack by DNA bases to form monoadducts and interstrand crosslinks, preferentially targeting CpG sequences. This mechanism demonstrates particular efficacy against upper gastrointestinal and bladder cancers (81, 159).

Additional *Streptomyces*-derived compounds demonstrate diverse mechanisms. Geldanamycin from *Streptomyces hygrocopicus* induces osteosarcoma cell death by depleting mitochondrial HSP60 pools while enhancing protein acetylation (160). Trioxacarcins from *Streptomyces bottropensis* showed activity against sarcoma and leukemia through covalent DNA binding and macromolecule synthesis inhibition (161–163). Salinomycin, isolated from *Streptomyces albus*, has garnered significant interest for its capacity to target both bulk tumor cells and CSCs, potentially reducing therapeutic resistance (68, 164, 165). Chromomycin A3 from *Streptomyces griseus* binds the DNA minor groove, inhibiting RNA synthesis with demonstrated promise in leukemia treatment (166, 167).

## 4 Technological innovations in NP exploration

Environmental bacteria represent considerable promise for the identification of novel anticancer drugs characterized by distinctive structures and unexplored modes of action. In this context, advances in metagenomics, genome mining, synthetic biology, and high-throughput screening have fundamentally transformed NP discovery from environmental bacteria. These approaches enable access to the vast chemical diversity encoded in bacterial genomes that remains inaccessible through conventional culture-based approaches. A summary of key technological advancements, including HTS, metagenomics, and synthetic biology, and their respective applications in anticancer NP discovery is provided in **Table 4**.

**Table 4:** Technological advancements in drug discovery: applications of HTS, metagenomics, and synthetic biology.

Technology	Examples of applications	Description of use in drug discovery	References
High-Throughput Screening (HTS)	Withaferin A (from *Withania somnifera*), Plinabulin (from marine *Aspergillus* sp.)	HTS allows rapid screening of thousands of compounds, identifying those with potential anticancer activity. Withaferin A targets prostate cancer, while Plinabulin disrupts microtubule dynamics in lung cancer cells and reduces chemotherapy-induced side effects.	(47, 168, 169)
Metagenomics	Salinosporamide A (from *Salinispora tropica*)	Metagenomics involves extracting DNA directly from environmental samples, enabling the discovery of compounds from unculturable organisms. Salinosporamide A, found using this approach, inhibits the 20S proteasome and is under clinical trials for treating multiple myeloma and glioblastoma.	(44, 48)
Synthetic Biology	Engineered biosynthetic pathways in bacteria to produce novel analogs of existing drugs, including modifications of anticancer agents like doxorubicin	Synthetic biology customizes biosynthetic pathways, enhancing yield and modifying structure to improve drug activity and selectivity. Engineered bacterial pathways allow for novel analog production, reducing toxicity and potentially enhancing efficacy in cancer treatment.	(45, 170, 171)

### 4.1 High-throughput screening

High-throughput screening (HTS) is an effective method that facilitates the rapid evaluation of several NPs across various targets simultaneously, accelerating the process of NP discovery. Using HTS, researchers can evaluate the effects of several compounds on diverse cancer cell lines at the same time, facilitating an assessment of each compound’s activity range and its potential influence on CSCs, which are frequently linked to resistance and recurrence (47). HTS facilitates the visualization of potential drug-drug interactions, leading to the identification of combination medicines that increase anticancer efficacy. This method has emerged as a fundamental aspect of NP discovery, as it considerably reduces the time needed to find potential compounds (42). The NCI-60 Human Tumor Cell Lines Screen, operational for over 30 years, has evaluated more than 110, 000 compounds and 50, 000 natural product extracts across 60 human tumor cell lines representing leukemia, lung, colon, brain, melanoma, ovarian, renal, prostate, and breast cancers (172). HTS platforms facilitate assessment of compound activity spectra, effects on CSCs implicated in resistance and recurrence, and potential synergistic drug combinations that enhance anticancer efficacy.

To address compatibility challenges of crude extracts with liquid handling systems and pan-assay interfering compounds, the NCI Program for Natural Product Discovery has created a prefractionated library of over 1 million fractions from 125, 000 NP extracts, representing perhaps the largest HTS-amenable natural product collection globally. Prefractionated extracts demonstrate enhanced biological activity through improved screening performance and reduced interference (173–175).

HTS has identified multiple promising anticancer compounds, including withaferin A (20) from *Withania somnifera*, which induces apoptosis and inhibits proliferation in breast and prostate cancer cell lines, and plinabulin from marine *Aspergillus* sp., which disrupts microtubule dynamics in non-small cell lung cancer while reducing chemotherapy-induced neutropenia (168, 176). Advanced HTS methodologies now incorporate three-dimensional cultures, cancer stem cell models, cell-based reporter assays targeting specific physiological pathways, and molecular assays directed at microtubules and protein kinases (176, 177).

### 4.2 Metagenomics, genomic exploration and synthetic biology

Metagenomics has revolutionized NP discovery by circumventing cultivation requirements, accessing the estimated 99% of environmental bacteria that remain unculturable under standard laboratory conditions (44, 152, 178). This approach involves direct extraction of environmental DNA from soil, water, or other samples, followed by sequencing to obtain complete metagenomes. Bioinformatics tools then identify biosynthetic gene clusters (BGCs) encoding natural product synthesis, which can be heterologously expressed to generate novel compounds *in vitro* (178, 179). Salinosporamide A (marizomib), discovered from the previously unculturable marine bacterium *Salinispora tropica* using metagenomic techniques, exemplifies this potential. This 20S proteasome inhibitor has advanced to clinical trials for multiple myeloma and glioblastoma (152).

Recent advances in next-generation sequencing, particularly PacBio HiFi technology, coupled with single-cell metagenomics and function-based screening, have expanded access to biosynthetic diversity from previously unstudied taxa and ecological niches. Phylogeny-guided metagenomic mining uses biosynthetic genes as molecular markers, with phylogenetic trees prioritizing BGCs for metabolite characterization based on evolutionary distance from characterized clusters (48, 179–181).

Complementing metagenomics, genome mining technologies enable direct identification and analysis of biosynthetic gene clusters (BGCs) directly from bacterial genomes, frequently revealing cryptic clusters activated only under specific conditions to generate novel compounds (170). Bioinformatic platforms including antiSMASH 7.0, DeepBGC, and GATOR-GC systematically discover hidden BGCs, particularly in under-explored taxa, and perform comparative genomic analyses to assess cluster conservation and diversity (180, 182–184).

Synthetic biology extends these capabilities by enabling BGC modification to optimize yield and adjust compound structures, improving both activity and selectivity against cancer targets (179, 180, 185). This includes the customization of biosynthetic pathways, permitting synthesis of novel derivatives with improved pharmacological characteristics through polyketide synthase and non-ribosomal peptide synthetase engineering (171). Notably, synthetic biology also enables the generation of “neo-natural products”, compounds not found in nature but produced by co-expressing diverse BGCs in novel combinations. This strategy falls within the broader framework of synthetic biology and frequently yields bioactivity superior to established drugs (45, 46, 152, 186). Multi-omics integration that combines genomic prediction, metabolomics analysis (ultra-sensitive HRMS High-Resolution Mass Spectrometry with resolution ≥100, 000), and experimental validation, constitutes the current “deep mining” paradigm, transitioning discovery from serendipitous isolation to data-driven, targeted approaches (180, 187, 188).

These methodologies, HTS, metagenomics, genome mining, and synthetic biology, embody the pinnacle of technical advancement in NP discovery, especially for anticancer compounds. Collectively, they facilitate exploration of previously inaccessible NPs, accelerate discovery timelines, and enhance prospects for identifying next-generation cancer therapeutics with unprecedented structural diversity and mechanistic novelty.

## 5 MENA-derived NPs with anticancer potential

**Figure 3:**
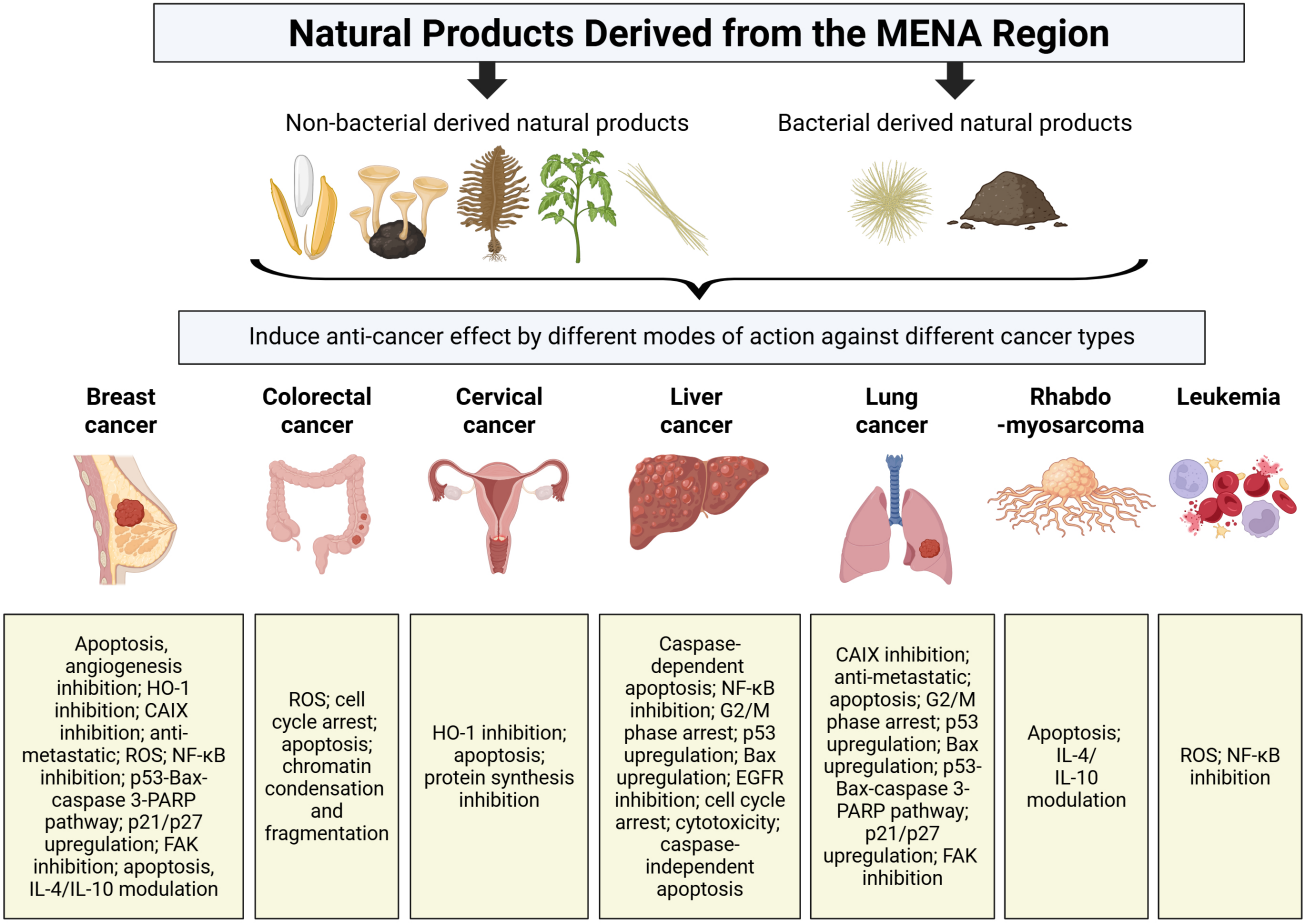
Natural products derived from the MENA region, including bacterial and non-bacterial sources, exert anticancer effects across multiple tumor types through diverse molecular mechanisms such as apoptosis induction, cell cycle arrest, ROS (reactive oxygen species) generation, and signaling pathway modulation. Created in BioRender. Bahmad, H (2026). https://BioRender.com/0gz7dwh.

Cancer remains a leading cause of global mortality despite advances in surgery, chemotherapy, radiotherapy, immunotherapy, and targeted therapies. Conventional treatments frequently encounter challenges including severe toxicities, acquired resistance, and limited efficacy in advanced or heterogeneous malignancies. These limitations have intensified interest in NPs, which offer structurally diverse bioactive compounds with multifaceted mechanisms including apoptosis induction, metastasis inhibition, and immune modulation (189, 190) (**Figure 3**).

In particular, the MENA region represents an underexplored yet exceptionally rich source of natural compounds, due to its unique biodiversity shaped by distinct geological, climatic, and ecological characteristics. This region encompasses three formally identified global biodiversity hotspots with high levels of endemism: the Mediterranean Basin, the Horn of Africa, and portions of the Eastern Afromontane. The Mediterranean Basin harbors around 5, 500 endemic plants out of a total of more than 25, 000 vascular plant species. This makes it the third most important hotspot in the world for plant diversity (191). In contrast, the Horn of Africa hotspot extending into Yemen, Oman, and the southern Arabian Peninsula, has 2750 endemic species out of approximately 5, 000 vascular plant species (192). This remarkable endemism is rooted in the region’s complex geological and paleogeographic history, including the separation of the Arabian Plate from Africa and the formation of Red Sea, events that isolated populations and drove speciation (193).

Beyond vascular plants, the MENA region harbors ecologically extreme environments that support highly distinctive microbial communities of equal relevance to natural product discovery. The Red Sea’s deep-sea anoxic brine pools, combining high salinity, extreme temperatures, heavy metals, and low oxygen, represent one of the most extreme marine environments on Earth, and their resident bacteria are considered promising candidates for novel bioactive molecules (194, 195). Metagenomic analysis of these brine pools has identified 2, 751 biosynthetic gene clusters spanning 28 classes, including polyketides, non-ribosomal peptides, and terpenes with predicted anticancer activities (7, 196–198). Bacterial extracts from these pools have further demonstrated direct pro-apoptotic effects against human cancer cell lines *in vitro* (199, 200). Terrestrial extreme environments contribute similarly, with actinobacterial communities from Algerian Saharan soils shown to develop novel metabolic pathways and yield new secondary metabolites under conditions of extreme heat and salinity (201). This enhanced chemical output under stress is due to abiotic stress, a recognized driver of secondary metabolite diversification (202).

The convergence of high taxonomic endemism, deep geological isolation, and persistent ecological extremes across the MENA region therefore provides a strong rationale for prospecting its flora and microbiome as sources of novel anticancer natural products.

The origin, active compound, and molecular and structural formulae of representative MENA-derived anticancer NPs discussed throughout this section are summarized in **Table 5**.

**Table 5:** MENA-derived natural products with anticancer potential.

Organism/source	Geographic origin	Extract/bioactive molecules	Major compound class	Cancer models	Key anticancer mechanisms	Reference(s)
*Jania rubens*	Egypt, Lebanon	Methanolic extract	Phenolics; terpenoids	Colorectal cancer	ROS modulation; cell cycle arrest	(201)
*Aspergillus fumigatus* (WA7S6)	Egypt (Red Sea sponge microbiome)	Fatty acids; dehydromevalonic lactone	Fatty acids; lactones	Breast; cervical cancer	HO-1 inhibition; apoptosis induction	(203)
*Aspergillus niger*	Egypt (Red Sea coast)	Organic acids (oxalic, citric acid derivatives)	Organic acids	Breast; liver; colon cancer	Oxidative stress modulation; cytotoxicity	(204)
*Sesamum indicum*	Egypt	Sesamin; sesaminol; methyl linoleate	Lignans; lipid derivatives	Hepatocellular carcinoma	Caspase-dependent apoptosis	(205)
*Aberia caffra*	Egypt	Aberiamine (alkaloid)	Alkaloids	Hepatocellular carcinoma	Caspase-mediated apoptosis	(205)
*Ajuga iva*	Morocco	Flavonoids; phenylpropanoids	Flavonoids; phenolics	Angiogenesis models	VEGFR2/EGFR modulation; anti-angiogenic activity	(206)
*Ephedra alata*	Saudi Arabia	Chlorogenic acid; apigenin	Phenolic acids; flavonoids	Breast; lung cancer	CAIX inhibition; anti-metastatic effects	(207)
*Nigella sativa*	Egypt	Thymoquinone	Quinone (benzoquinone-type)	Leukemia; colorectal; breast cancer	NF-κB inhibition; ROS modulation; apoptosis	(208, 209)
*Withania somnifera*	Egypt, Sudan	Withaferin A	Withanolides (steroidal lactones)	Hepatocellular; breast cancer	NF-κB inhibition; apoptosis induction	(210–212)
*Solenostemma argel*	Sudan, Egypt	Flavonoid-rich extract	Flavonoids	Colorectal cancer	Apoptosis; DNA fragmentation	(213)
*Artemisia herba-alba*	Morocco, Algeria	Flavonoids; essential oils	Flavonoids; terpenoids	Hepatocellular; lung cancer	p53 activation; Bax upregulation; G2/M arrest	(214, 215)
*Punica granatum*	Lebanon, Egypt	Ellagic acid; polyphenols	Phenolics; flavonoids	Liver cancer	EGFR pathway inhibition	(216, 217)
*Inula viscosa*	Lebanon, Palestine	Lupeol; betulin; caryophyllene oxide	Terpenoids	Lung cancer	p53–Bax–caspase-3–PARP signaling; FAK inhibition	(218)
*Callyspongia siphonella*	Saudi Arabia	Sesterterpenoids	Terpenoids	Breast; liver cancer	Cell cycle arrest; apoptosis	(219)
*Dendronephthya hemprichi*	Egypt (Red Sea)	Crude extract (terpenoid-rich)	Terpenoids	Hepatocellular carcinoma	Apoptosis; caspase-independent cell death	(220)
*Sarcophyton glaucum*; *Sargassum* spp.	Egypt (Red Sea)	Crude extracts	Terpenoids; phenolics	Hepatocellular carcinoma	Cytotoxicity; anti-proliferative activity	(221)
*Actinokineospora* sp. EG49	Egypt (Red Sea sponge)	Actinosporins C & D	Aromatic polyketides	HL-60 leukemia cells	DNA protection; antioxidant activity	(221)
*Moorea producens*	Egypt; Saudi Arabia	Lyngbyatoxin A; debromoaplysiatoxin	Polyketides; cyclodepsipeptides	Cervical cancer	Protein kinase C modulation; protein synthesis inhibition	(221)
Unidentified actinomycete spp.	Egypt (Mediterranean coast)	Polyketides; cyclic peptides	Polyketides; peptides	HepG2; MCF-7; HCT-116	Cytotoxicity; apoptosis induction	(222)
*Serratia marcescens*	Iraq (soil)	Prodigiosin	Tripyrrole alkaloid	Breast; rhabdomyosarcoma	Mitochondrial dysfunction; apoptosis	(223)
*Streptomyces* sp. MS1B15	Egypt (soil)	Cyclic peptides; macrolides	Polyketides; peptides	Breast cancer	ROS modulation; anti-inflammatory effects	(224)
*Streptomyces* sp. AGM12-1	Egypt (soil)	Diketopiperazines	Cyclic dipeptides	Hepatocellular; colon cancer	Cytotoxicity; PKS/NRPS biosynthetic activity	(225)

### 5.1 Terrestrial arid systems and biogeographically structured phytochemical innovation

In MENA’s arid and semi-arid areas, plants are subjected to chronic, multi-axial stressors like water scarcity, intense UV light, soil salinity, and extreme thermal fluctuation. Unlike temperate regions where such stresses are intermittent, these conditions are constitutive across MENA ecosystems, exerting sustained selective pressure on secondary metabolite biosynthesis and influencing the development of beneficial phytochemicals (226).

For example, *Ephedra alata*, endemic to the saline zones of Saudi Arabia, exhibited cytotoxic activity against breast and lung cancer cell lines via compounds like chlorogenic acid and apigenin. These same compounds were predicted to interact with hypoxia-associated molecular targets (207). Moreover, *Artemisia herba-alba* induced G2/M cell-cycle arrest and apoptosis via p53 activation (214, 215), while *Solenostemma argel* demonstrated cytotoxicity against colorectal cancer through standard apoptotic pathways (213). These activities reflect evolutionarily conserved biosynthetic adaptations rather than incidental chemical diversity, arising from prolonged exposure to conditions that parallel the oxidative and genotoxic stresses encountered in malignant transformation.

In Mediterranean-North African transition zones, phytochemical diversification is further shaped by overlapping biogeographic conditions. *Ajuga iva* from Morocco exerts anti-angiogenic effects through modulation of EGFR and VEGFR2 signaling (206). *Sesamum indicum* and *Aberia caffra* from Egypt demonstrate hepatocellular cytotoxicity, driven by their phenolic and lipid-based compounds (205). This chemical convergence across African-tropical and Mediterranean plant life reflects shared biosynthetic responses to analogous environmental pressures rather than phylogenetic proximity.Ethnomedicinal plants like *Nigella sativa* and *Withania somnifera* further illustrate the therapeutic and pharmacological relevance of MENA traditional medicine. Their principal bioactive compounds, thymoquinone and withaferin A, share similar oncogenic signaling networks, including NF-κB, STAT3, Akt/mTOR, and Hsp90 systems, modulating tumor survival and progression (208, 227, 228).

The conservation of these targets across diverse MENA plant lineages underscores the therapeutic coherence of the region’s stress-adapted phytochemical repertoire.

Terrestrial venoms of the MENA region, produced by scorpions and vipers endemic to its landscapes, represent an additional and largely underexplored but chemically sophisticated reservoir of anticancer bioactive molecules. These venoms are complex mixtures of bioactive peptides and proteins, shaped by long-term evolutionary adaptation to arid environments and resource-scarce, predator-prey dynamics characteristic of desert ecosystems. Their constituent molecules interact with tumor-relevant targets with high selectivity inhibiting proliferation, inducing apoptosis, suppressing angiogenesis, and attenuating metastatic dissemination through modulation of chloride channels, matrix metalloproteinases, and membrane depolarization pathways (209, 229).

One of the most extensively investigated venom-derived peptides is chlorotoxin, originally isolated from *Leiurus quinquestriatus*. Chlorotoxin binds glioma-associated chloride channels and MMP-2 complexes with high affinity, establishing a mechanistic basis for its development as both a tumor-targeting diagnostic agent and a therapeutic delivery platform.The structural diversity of scorpion venom encompassing ion channel modulating peptides and cell penetrating peptides, further support its exploitation as a scaffold for designing targeted drug delivery systems in cancer care (230–232).

Snake venoms from MENA vipers represent a pharmacologically rich source of disintegrins, phospholipase A_2_ enzymes, and metalloproteinases. These molecules interfere with integrin–extracellular matrix interactions, disrupt tumor cell adhesion and signaling, and trigger apoptotic cascades with relative selectivity for malignant over non-malignant cells. Preclinical evidence additionally supports antiangiogenic, antiproliferative, and immunomodulatory activities, positioning these venom fractions as candidates for rational therapeutic development. Advances in peptide engineering and recombinant expression technologies have facilitated the transition from crude venom bioactivity screening to structure guided therapeutic development (233–236).

Beyond their direct cytotoxic properties, MENA terrestrial venoms are of broader scientific significance as products of evolutionary optimization under the same arid, thermally extreme conditions that characterize the region’s biodiversity hotspots, making their chemical repertoire both ecologically distinctive and pharmacologically relevant.

Nevertheless, most venom-derived anticancer candidates remain in preclinical development stages. Translational advancement is delayed by systemic toxicity profiles, unfavorable pharmacokinetic parameters, immunogenicity, and the technical challenges of precise tissue-targeted delivery (233, 237, 238). Bridging these gaps will require systematic application of venomics, transcriptomics, and structural bioengineering frameworks to enable rigorous mechanistic characterization and clinical translation (239–241). Key venom-derived molecules, their sources, anticancer mechanisms, and developmental status are compiled in **Table 6**.

**Table 6:** MENA venom-derived bioactive molecules with anti-cancer potential.

Venom source	Geographic origin	Bioactive molecules	Molecular class	Cancer models/targets	Key anticancer mechanisms	Reference(s)
*Leiurus quinquestriatus* (Scorpion)	MENA (North Africa, Egypt region)	Chlorotoxin	Disulfide-rich peptide neurotoxin	Glioma and solid tumor targeting models	Binding to glioma-associated chloride channels and MMP-2 complexes; tumor cell invasion inhibition; tumor-targeted delivery platform	(230, 231)
Scorpion venoms (general MENA species)	MENA region	Ion channel–modulating peptides	Neurotoxic peptides	Breast cancer; leukemia; solid tumor cell lines	Induction of apoptosis; cell cycle arrest; inhibition of migration and angiogenesis via ion channel modulation	(65, 232, 237, 240)
Scorpion venom peptides	MENA region	Diverse bioactive peptides	Peptides (ion channel modulators, CPPs)	Multiple cancer cell lines (*in vitro*)	Membrane depolarization; modulation of chloride and potassium channels; apoptosis induction; suppression of tumor growth signaling	(231, 232, 242)
Scorpion venom–derived compounds	MENA region	Venom protein fractions	Proteins and peptide mixtures	Breast cancer cell lines	Regulation of inflammatory signaling; apoptosis induction; ROS-mediated cytotoxicity	(238, 242)
*Cerastes cerastes* (Viper)	Saudi Arabia/Arabian Peninsula	Phospholipase A_2_ (PLA_2_) isoforms	Secreted enzymatic toxins	Breast cancer; tumor cell lines (*in vitro*)	Membrane phospholipid hydrolysis; apoptosis induction; disruption of tumor cell signaling and viability	(233, 235)
MENA viper venoms (general)	Middle East & North Africa	Disintegrins	Cysteine-rich peptides	Solid tumor metastasis models	Inhibition of integrin–ECM binding; anti-angiogenic effects; suppression of tumor invasion and metastasis	(233, 234)
MENA snake venoms (general)	Middle East & North Africa	Snake venom metalloproteinases (SVMPs)	Zinc-dependent proteases	Tumor progression models	ECM degradation modulation; inhibition of tumor invasion; anti-angiogenic and pro-apoptotic effects	(233, 234)
Scorpion and arthropod venoms (engineering studies)	MENA-derived species	Venom-derived peptides	Bioactive peptides	Drug delivery and nanomedicine cancer systems	Cell-penetrating activity; tumor targeting; nanocarrier functionalization; enhanced drug delivery specificity	(239)
Venom peptide libraries (systematic studies)	MENA region	Multi-component venom fractions	Proteomic peptide mixtures	Breast cancer and leukemia models	Multi-target modulation including NF-κB suppression, apoptosis activation, and immune signaling regulation	(238, 240, 242)

The primary significance of MENA terrestrial flora and fauna lies not in the novelty of chemical scaffolds alone, but in demonstrating how persistent multi-axial environmental stressors drive conserved yet pharmacologically relevant biosynthetic trajectories across diverse organisms.

### 5.2 Marine systems of the Red Sea and Eastern Mediterranean as evolutionarily isolated chemical laboratories

The Red Sea and Eastern Mediterranean have unique marine ecosystems, with semi-enclosed and geographically isolated marine basins characterized by extreme salinity, elevated thermal gradients, and high irradiance. These conditions distinguish them fundamentally from open Indo-Pacific systems and impose persistent selective pressures that have shaped chemically distinctive marine biota (243).

For instance, *Jania rubens*, a calcareous red alga distributed across the Eastern Mediterranean, exhibited cytotoxic, antioxidant, and anti-migratory activities linked to an enriched complement of phenolic compounds and flavonoids. This accumulation reflects biosynthetic upregulation under chronic UV and hypersaline stress (244).

Red Sea sponge-associated fungi, like *Aspergillus fumigatus*, produce bioactive secondary metabolites under conditions of persistent hypersalinity and thermal stress. Rather than functioning as isolated microbial producers, these organisms operate within host-structured symbiotic chemical systems, in which metabolite expression is shaped by persistent hypersaline and thermal selection pressures acting on fungal host interactions (203).

Marine invertebrate holobionts, like *Callyspongia siphonella*, produce bioactive compounds that disrupt mitochondrial membrane integrity, activate apoptotic cascades, and suppress cancer cell migration (219). Soft corals, sponges, and macroalgae from the Red Sea collectively exhibit substantial chemodiversity, reflecting their prolonged evolutionary isolation and adaptation to one of the world’s most physiochemically extreme marine environments (220).

Researchers are now more focused on using ecology-driven discovery methods for studying the Red Sea and Eastern Mediterranean marine ecosystems, rather than just looking for new compounds. Despite their chemical richness, the translational development of Red Sea and Eastern Mediterranean marine NPs remains at an early stage. The majority of studies have not progressed beyond crude extract bioactivity screening, with few advancing to full structural elucidation or robust *in vivo* validation. Mechanistic evidence remains limited, and reliance on standard cytotoxicity endpoints restricts the capacity to predict clinical relevance (245).

Future research should concentrate on integrated metabolomics, genome mining, and functional pharmacology as complementary frameworks for advancing structurally defined marine NPs toward preclinical and translational development. The field is shifting from extract-based bioactivity surveys toward ecology-driven discovery paradigms that incorporate symbiosis, environmental selection, and biosynthetic gene cluster analysis as primary analytical frameworks.

### 5.3 Extreme microbial ecosystems as stress-integrated biosynthetic evolution systems

Microbial ecosystems across the MENA region form complex systems defined by the convergence of extreme environmental parameters like salinity, aridity, hypoxia, and metal exposure. These factors are not only drivers of taxonomic diversity, but are also selective forces that promote diversification of biosynthetic gene clusters encoding NRPS, PKS, and hybrid metabolic pathway (246).

In the Red Sea’s Atlantis II Deep basin, a comparable pattern is observed. This geochemically isolated hydrothermal system is characterized by sustained physicochemical extremity harboring microbial communities with markedly enriched biosynthetic gene cluster repertoires. Metagenomic analysis has identified NRPS, radical SAM, and glycosyltransferase-related pathways encoding putative anticancer metabolites (247). Notably, genome-mining approaches enable bioactivity prediction directly from sequence data, circumventing the cultivation barrier that makes the majority of this microbial diversity inaccessible through conventional approaches (248).

Marine sponges and cyanobacteria from the region contribute a structurally diverse repertoire of bioactive metabolites, like actinosporins and lyngbyatoxins, with documented cytotoxic and signaling modulatory activities (219). Sponge-associated fungi such as *Aspergillus fumigatus* and *A. niger*, produce fatty acid derivatives and organic acids that modulate oxidative stress pathways in cancer cells (219). This biosynthetic diversity reflects co-evolutionary adaptation among microbial communities, their hosts, and the extreme physicochemical environments they inhabit.

Actinomycetes, especially Streptomyces species isolated from MENA mangroves, coastal sediments, and desert soils, produce numerous cyclic peptides, polyketides, and macrolides with selective cytotoxicity across multiple cancer types (222–225). Mangrove-coastal interfaces represent biosynthetic hotspots where the convergence of marine and terrestrial stress regimes drives BGC diversification beyond that observed in comparable non-arid environments globally.

Terrestrial bacteria like *Serratia marcescens* produces prodigiosin, a tripyrrole alkaloid that induces apoptosis through DNA intercalation and mitochondrial membrane disruption. Soil-dwelling Streptomyces species produce polyketides and non-ribosomal peptides with selective toxicity against cancer cells. In the harsh Sahara-Arabian environment, these microbes thrive under unique stress conditions, leading to distinct biosynthetic products (223).

Collectively, MENA microbial ecosystems demonstrate that anticancer metabolite diversity is not simply a product of environmental extremity, but of the specific evolutionary trajectories imposed by regionally distinct stress regimes acting on biosynthetic networks over geological timescales. Genome mining predictions within these systems frequently outpace experimental confirmation, emphasizing the need for integrated functional genomics and pharmacological validation before their translational potential can be fully realized.

## 6 Conclusion and future perspectives

The MENA region harbors a chemically diverse and largely underexplored repertoire of medicinal flora, fauna, and microbiota, shaped by the intersection of extreme ecological pressures and millennia of ethnopharmacological practice. This review demonstrates that MENA derived natural products, including alkaloids, flavonoids, terpenoids, and polyphenols, exhibit documented cytotoxic, antiproliferative, pro-apoptotic, and anti-metastatic activities across various cancer models (249). Integration of ethnobotanical knowledge with contemporary pharmacological and genomic screening has accelerated the identification of bioactive scaffolds with genuine therapeutic promise.

Natural products have contributed substantially to cancer drug development, with approximately 84% of the 185 small-molecule anticancer agents approved between 1981 and September 2019 being classified as natural products, natural product derivatives, or synthetics structurally inspired by natural product scaffolds (2, 38, 49, 51). Plant-derived agents including vincristine, vinblastine, paclitaxel, and topotecan exemplify successful clinical translation, while marine and microbial sources continue to expand the structural diversity of candidates entering development pipelines.

Despite this progress, significant challenges persist in natural product drug development. Key obstacles include compositional variability in crude extracts, limited clinical evaluation, poor bioavailability, low solubility, and drug resistance. Biphasic dose-response effects have been documented for several compounds, where sublethal concentrations paradoxically promote cancer cell proliferation rather than inhibition (250). Additionally, sustainable harvesting practices, biodiversity conservation, and intellectual property protection remain critical unresolved concerns (250–252).

Addressing these challenges necessitates coordinated efforts among regional research institutions, international collaborators, and pharmaceutical stakeholders. Implementation of Good Agricultural and Collection Practices (GACP), sustainable cultivation in controlled environments, and development of standardized protocols can ensure genetic integrity and consistent bioactive compound profiles. Advanced approaches including multi-omics technologies, nanotechnology-based drug delivery systems, and semi-synthetic modifications offer promising strategies to overcome bioavailability limitations and enhance therapeutic efficacy.

MENA natural products represent a promising yet underexplored frontier for anticancer drug discovery. Sustained investment in mechanistic research, translational infrastructure, and ethical governance frameworks will be essential to converting the region’s exceptional chemical diversity into clinically viable therapeutics with global relevance.
